# IGF-IR Mediated Mammary Tumorigenesis Is Enhanced during Pubertal Development

**DOI:** 10.1371/journal.pone.0108781

**Published:** 2014-09-26

**Authors:** Robert A. Jones, Katrina L. Watson, Craig I. Campbell, Roger A. Moorehead

**Affiliations:** 1 Department of Biomedical Sciences, Ontario Veterinary College, University of Guelph, Guelph, Ontario, Canada; 2 Lunenfeld-Tanenbaum Research Institute, Mount Sinai Hospital, Joseph & Wolf Lebovic Health Complex, Toronto, Ontario, Canada; Insamlingsstiftelsen Adamovic Cancer Research; Adamovic Research AB, Sweden

## Abstract

Although breast cancer typically develops in women over the age of 40, it remains unclear when breast cancer initiating events occur or whether the mammary gland is particularly susceptible to oncogenic transformation at a particular developmental stage. Using MTB-IGFIR transgenic mice that overexpress type I insulin-like growth factor receptor (IGF-IR) in a doxycycline-inducible manner, mammary tumorigenesis was initiated at different developmental stages. Tumor multiplicity was significantly increased while tumor latency was significantly decreased when the IGF-IR transgene was expressed during pubertal development compared to post-pubertal transgene expression. Moreover, metastatic spread of mammary tumors to the lungs was approximately twice as likely when IGF-IR was overexpressed in pubertal mice compared to post-pubertal mice. In addition, engraftment of pubertal MTB-IGFIR mammary tissue into cleared mammary fat pads of pubertal hosts produced tumors more frequently and faster than engraftment into adult hosts. These experiments show that the mammary microenvironment created during puberty renders mammary epithelial cells particularly susceptible to transformation.

## Introduction

The mammary gland is a complex secretory organ that requires the interaction of multiple cell types for normal development and function. The two major cell lineages within the mammary epithelium are luminal cells that line the inner layer of the mammary ducts and an outer layer of contractile myoepithelial cells. In addition, a diverse array of stromal cells including adipocytes, vascular endothelial, fibroblasts and immune cells constitute the mammary fat pad in which the ductal epithelial network is embedded [1]–[3].

Mammary gland development can be divided into distinct stages that begin during embryogenesis and continue throughout reproductive maturity and adulthood. In mice, multipotent fetal mammary stem cells give rise to a rudimentary bilayared ductal tree that remains quiescent until the onset of puberty. During the pubertal phase, increased levels of circulating ovarian hormones coincide with the appearance of highly proliferative structures called terminal end buds, which drive exponential growth and branching of the ductal system. Pregnancy also results in dramatic morphological changes characterized by the rapid proliferation and differentiation of epithelial cells into lobuloalveoli in preparation for lactation [1]–[4]. Following cessation of milk production, involution leads to the collapse of lobuloalveoli and returns the mammary gland to the pre-pregnant state. In contrast, the virgin adult mammary gland is often considered as ‘resting’ and being in an inactive state. However, mammary tissue undergoes pregnancy-like development during estrous that is associated with limited periods of proliferation and involution. Thus, the mammary gland is a unique and highly dynamic organ that experiences morphological and cellular changes that span prenatal and postnatal stages of life [1]–[4].

Although most breast cancers develop in women after the age of 40, it remains unclear when the actual breast cancer initiating events occur. Evidence from human and rodent studies indicate that the mammary gland is particularly susceptible to transformation during pubertal development while completion of a full term pregnancy protects against breast cancer development. Epidemiologic data has shown that late menarche and an early first term pregnancy are associated with a decreased risk of developing breast cancer [5]. In fact, the only known factor to consistently decrease lifetime breast cancer risk regardless of ethnic background is early childbirth [5], [6]. Although the exact mechanisms underlying the protective effects of pregnancy remain unclear, it has been hypothesized that pregnancy leads to the depletion of the progenitor cell population resulting in a more differentiated environment that is less susceptible to transformation [7]–[11]. Additional evidence suggesting that the pubertal mammary gland is particularly sensitive to transformation includes a study that examined breast cancer rates in Japanese atomic bomb survivors. Women younger than 20 years of age at the time of radiation exposure have a 13-fold elevated risk of developing breast cancer before the age of 35 and a 2-fold elevated lifetime risk of developing breast cancer compared to women who were over 35 years of age at the time of exposure [12]. In addition, breast cancer is the most common secondary lesion in pubertal females treated with chest X-ray for Hodgkin’s lymphoma [13]–[16]. Rodent studies also suggest that the mammary gland is particularly susceptible to transformation during pubertal development. Rats exposed to chemical carcinogens such as 7,12-dimethylbenz [a]anthracene (DMBA) or N-methyl-N-nitrosourea (NMU) during puberty, but not following a full term pregnancy, developed mammary tumors [17]–.

Most of the research related to breast cancer has focused on alterations within the mammary epithelium. Chemicals, radiation or other carcinogenic agents have been postulated to induce mutations that provided a proliferative and survival advantage [22], [23]. However, in 1963 James Orr proposed that alterations in the stromal environment could also influence tumorigenesis [24]. The importance of the stromal compartment in regulating epithelial cell fate was demonstrated in cell recombination experiments. Co-culture of mammary epithelial cells with mammary-derived mesenchyme produced ductal branching similar to that observed in the mammary gland while co-culture with salivary mesenchyme led to repeated bifurcation of terminal buds resulting in epithelial structures that resembled salivary epithelium [25], [26]. These studies highlight the organ specific nature of mesenchyme function and the importance of the stroma in directing proper epithelial morphogenesis.

The stromal environment has also been implicated in regulating mammary tumorigenesis. Studies in rats showed that recombining normal mammary epithelial cells with stromal cells that have been exposed to carcinogens or radiation can promote tumor initiation and progression [27], [28] while normal mouse stroma induced differentiation of murine mammary adenocarcinoma cells [29]. In addition, injection of human breast cancer cells mixed with normal mammary epithelial cells into mammary fat pads of nude mice resulted in the generation of a normal mammary ductal tree [30]. It has been hypothesized that normal mammary stroma can suppress mammary tumor progression by provoking a more differentiated state [29], [31].

To evaluate whether the mammary stromal environment could influence tumor formation, mammary tumors were initiated at different developmental stages in MTB-IGFIR transgenic mice. MTB-IGFIR transgenic mice express elevated levels of the type I insulin-like growth factor receptor (IGF-IR) specifically in mammary epithelial cells in a doxycycline-inducible manner. Overexpression of IGF-IR in mammary epithelial cells leads to the development of mammary tumors that share features with human basal-like breast cancer [32]. Since expression of the IGF-IR transgene is dependent on the presence of doxycycline, the impact of IGF-IR overexpression on cellular transformation at different developmental stages can be investigated [32]. Using this model, we observed that the mammary gland is most susceptible to IGF-IR-induced transformation during pubertal phase of mammary gland development. Furthermore, tissue recombination experiments revealed the microenvironment of the pubertal gland enhances mammary tumorigenesis. Taken together these findings suggest that oncogenic transformation is influenced by the developmental state of the mammary gland and these effects are mediated by the stromal microenvironment.

## Materials and Methods

### Ethics

Animals were housed and cared for following guidelines established by the Central Animal Facility at the University of Guelph and the guidelines established by the Canadian Council of Animal Care. This study was approved by the Animal Care Committee at the University of Guelph (AUP# 1695) and all efforts were made to minimize suffering.

### Mice

MTB-IGFIR transgenic mice were generated in our lab and have been previously described [32]. Expression of the IGF-IR transgene was induced in mammary epithelial cells with rodent chow supplemented with 2 g/kg doxycycline (Harlan Laboratories, Mississauga, ON, Canada) beginning when the offspring’s mothers were mated (embryonic) at postnatal day 21 (pubertal), at postnatal day 100 (adult) or after a full pregnancy and lactation cycle (primiparous). All mice were monitored for tumor development at least 2 times per week by palpation. Once the mammary tumors reached either 17 mm in diameter or 10% of the mouse’s body weight, mice were euthanized and the mammary tumors were divided for fixation in 10% neutral buffered formalin or flash frozen for molecular analysis.

### Western Blotting

Western blotting was performed as described in Jones et al [32]. All antibodies were obtained from Cell Signalling Technologies (Beverly, MA) except for IGF-IR which was obtained from R&D Systems (Minneapolis, MN) and β-actin antibody which was obtained from Sigma (Oakville, ON). All antibodies were used at a 1∶1,000 dilution except for β-actin which was used at a 1∶5,000 dilution. Appropriate secondary antibodies were obtained from Cell Signalling Technologies (Beverly, MA) and used at a dilution of 1∶2,000. Images were captured on a FluorChem 9900 gel documentation system (Alpha Innotech, San Leandro, CA) and quantification of western blots was performed using AlphaEase software (Alpha Innotech, San Leandro, CA).

### Histology and Immunohistochemistry

Mammary tumors and lungs were collected and processed as previously described [32], [33]. Immunohistochemistry was performed as previously described [32]. The number of lung metastases was determined in two independent histological sections from each mouse and then averaged. Primary antibodies were used at a dilution of 1∶200 and were obtained from the following sources, anti-Ki67, anti-cytokeratin 5 and anti-cytokeratin 14 (Abcam, Cambridge, MA), anti-cytokeratin 18 (Research Diagnostics Inc, Flanders, NJ), and anti-cytokeratin 8 (Fitzgerald Industries International Inc, Concord, MA). Primary antibodies were detected using a 1∶200 dilution of the appropriate secondary antibody and Sigma Fast 3,3′-diaminobenzidine tablets (Sigma, St. Louis, MO). Ki67 immunohistochemistry was quantified using Positive Pixel Count software v9 (Aperio, Vista, CA) following slide scanning on a ScanScope CS slide scanner (Aperio, Vista, CA).

### Statistics

IBM SPSS Statistics software was used to generate the Kaplan-Meier tumor free curves and the Breslow (Generalized Wilcoxon) statistic. An ANOVA followed by a Tukey’s test was used to determine statistical differences in tumor number. To compare the percentage of mice with lung metastases, a Fisher’s exact test was performed. Values were considered statistically significant when p<0.05.

## Results

### Mammary Tumor Development Is Delayed in Adult and Primiparous Mice

To investigate whether the developmental state of the mammary gland modulates mammary tumorigenesis, the IGF-IR transgene was induced during embryonic development, on postnatal day 21, on postnatal day 100 and after one complete cycle of pregnancy and lactation (primiparous). Since pubertal mammary development begins at approximately 4–5 weeks of age in FVB mice and is completed by approximately 8–10 weeks of age, expressing the IGF-IR transgene at postnatal day 21 ensures that the transgene is elevated during pubertal development while expressing the IGF-IR transgene at postnatal day 100 ensures that pubertal mammary development has been completed prior to IGF-IR overexpression.

**Figure 1:**
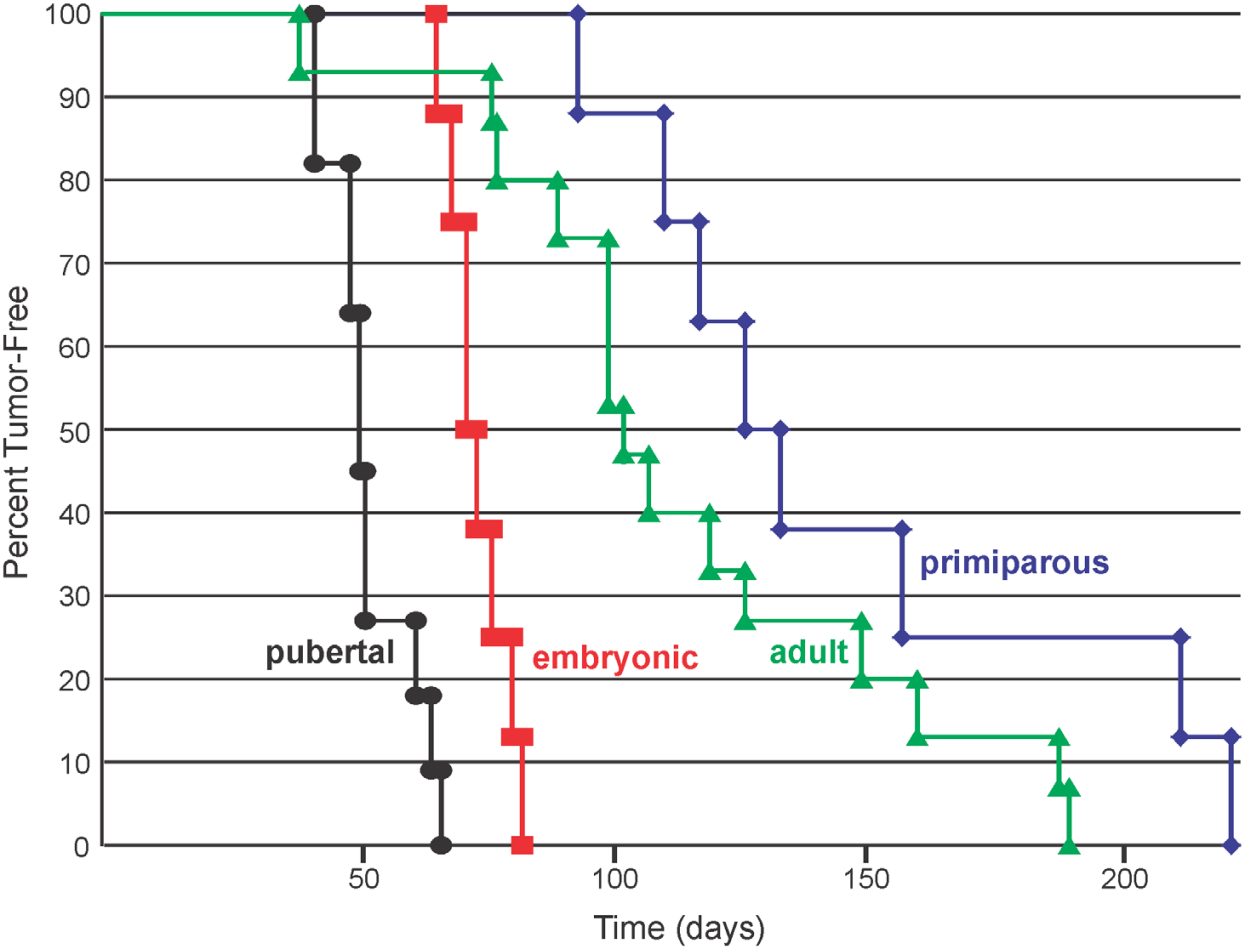
Kaplan-Meier plot showing the percentage of tumor-free mice at different time points after the IGF-IR transgene was induced by the addition of doxycycline to the animals’ food. The IGF-IR transgene was induced during embryonic development (red line), at 21 days of age (black line), at 100 days of age (green line), or after completion of one cycle of pregnancy and lactation (blue line). Tumor onset was significantly faster when the IGF-IR transgene was induced at day 21 of age compared to the other 3 groups as determined by Breslow (Generalized Wilcoxon) analysis. This statistical test also determined that tumor onset was faster in mice with IGF-IR induction during embryonic development compared to IGF-IR induction at 100 days of age or following pregnancy and lactation.

**Figure 2:**
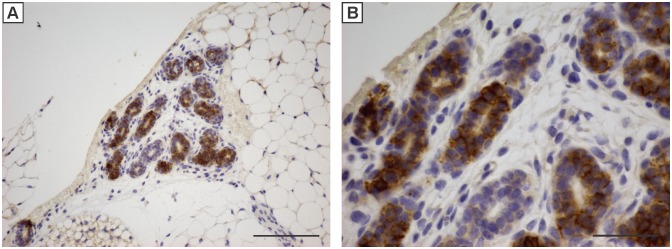
Immunohistochemistry for the IGF-IR (brown stain) in mammary epithelial cells of a 2 day old female mice captured at 100x (A) or 200x (B) magnification. Scale bars, 200 µM (A) and 100 µM (B).

Kaplan-Meier curves presented in Figure 1 show tumor onset following IGF-IR transgene induction at each developmental stage. To calculate tumor onset for the embryonic group, it was assumed that IGF-IR was expressed in mammary epithelial cells by at least postnatal day 1. This assumption was based on the following, (i) mammary epithelial cells arise around embryonic day 12.5–18.5, (ii) doxycycline has been shown to cross the placenta and can be transferred to breast milk (http://www.drugs.com/pregnancy/doxycycline.html and [34]), and (iii) elevated expression of IGF-IR could be detected in mammary epithelium from postnatal day 2 mice; the earliest time point examined (Fig. 2). Therefore, using postnatal day 1 as the starting point for IGF-IR induction may slightly underestimate tumor onset for mice in the embryonic group.

Tumor onset was significantly accelerated when IGF-IR was overexpressed beginning on day 21 (pubertal development) compared to all other stages. Tumor formation was also significantly faster in mice overexpressing IGF-IR during embryonic development compared to mice where IGF-IR overexpression was initiated in adult or primiparous mice (p<0.05). Although pregnancy has been shown to have a protective role in breast cancer, we only observed a small, nonsignificant delay (p = 0.112) in tumor onset in primiparous mice compared to virgin adult animals.

Tumor multiplicity was also influenced by specific stages of mammary gland development. IGF-IR overexpression during embryonic or pubertal phases resulted in increased tumor multiplicity compared to adult and primiparous mice (Table 1). The average number of mammary tumors in mice where IGF-IR expression was induced during embryonic development or on postnatal day 21 were 6.8±0.6 and 7.2±0.6, respectively while adult and primiparous animals developed less than 2 tumors per mouse.

**Table 1:** Tumor Characteristics Following IGF-IR Induction at Different Developmental Stages.

	Embryonic	Pubertal	Adult	Primiparous
Tumor Onset^1^	74.2±2.1^b^	52.9±2.5	115.5±10.9^a^^,^ b	147.2±16.8^a^^,^ b
Number of Tumors	6.8±0.6	7.2±0.6	1.8±0.2^a^^,^ b	1.2±0.2^a^^,^ b
Mice with Lung Metastases	7/10 (70%)	4/11 (36%)	2/9 (22%)	2/8 (25%)
Number of Metastases/Mouse^2^	1.6±0.4	2.5±1.2	2±1.0	1.8±0.8

1 measured as days after IGF-IR induction through the addition of doxycycline.

2 reflects only mice with lung metastases.

a significantly different from embryonic, p<0.05.

b significantly different from pubertal, p<0.05.

**Figure 3:**
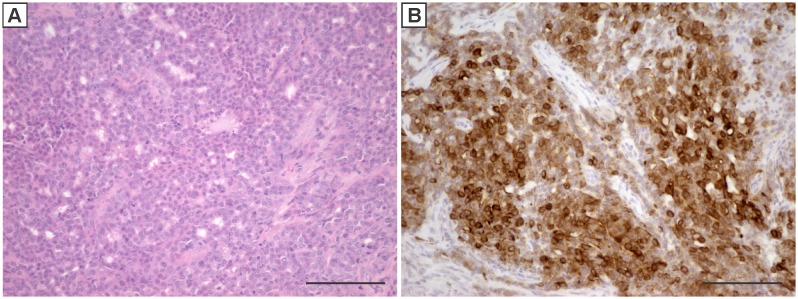
A representative H&E (A) stained section of a mammary tumor that develops resulting from IGF-IR induction in mammary epithelial cells. Immunohistochemistry for cytokeratin 8 (B) shows that most of the cells express this marker of luminal epithelial cells. Scale bars, 100 µM.

**Figure 4:**
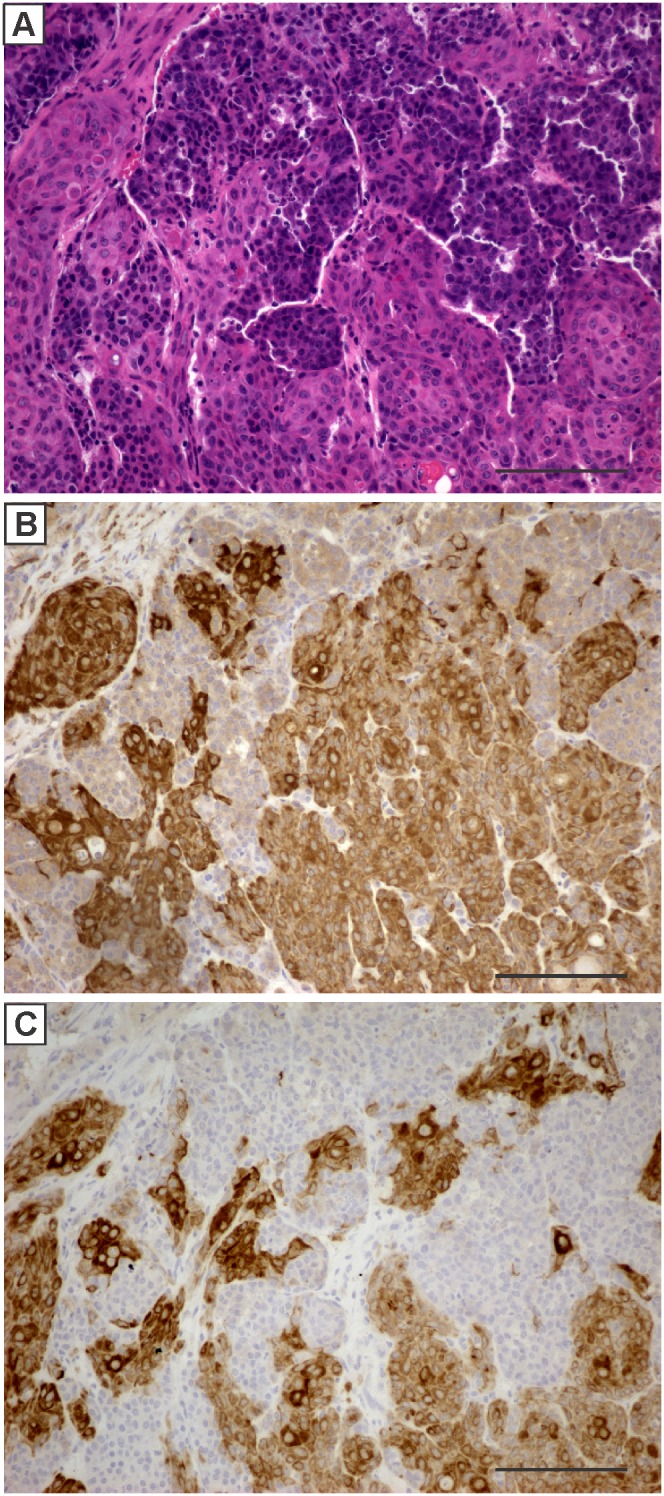
A representative H&E (A) stained section showing a region of a mammary tumor with squamous metaplasia. Cells in this region stain positive for the basal cell markers cytokeratin 5 (B) or cytokeratin 14 (C). Scale bars, 100 µM.

Histologically, the majority of tumors from all 4 groups were composed primarily of tumor cells with an epithelial morphology (Fig. 3A) that stained positive for cytokeratin 8 (Fig. 3B). Almost all of the tumors contained regions of squamous differentiation (Fig. 4A) as indicated by positive staining for cytokeratin 5 (Fig. 4B) or cytokeratin 14 (Fig. 4C). The extent of squamous differentiation was greater in the virgin mice compared to the primiparous mice. Using a cut-off of approximately 5% of the cells staining positive for either cytokeratin 5 or cytokeratin 14, all of the virgin mice had at least 30% of their tumors exceeding this cut-off while none of the primiparous mice had tumors with greater the 5% of the cells staining positive for either cytokeratin 5 or 14.

Previous studies with the MTB-IGFIR transgenic mice revealed that approximately 40% of the mice develop lung metastasis when the IGF-IR transgene is expressed during pubertal development [35]. Consistent with this observation, the current study found that 36% of the pubertal mice developed lung metastases. There was a trend towards decreased lung metastases in the adult and primiparous groups but this difference was not statistically significant (Table 1). There was also no statistical difference in the number of lung metastases that developed per mouse.

**Figure 5:**
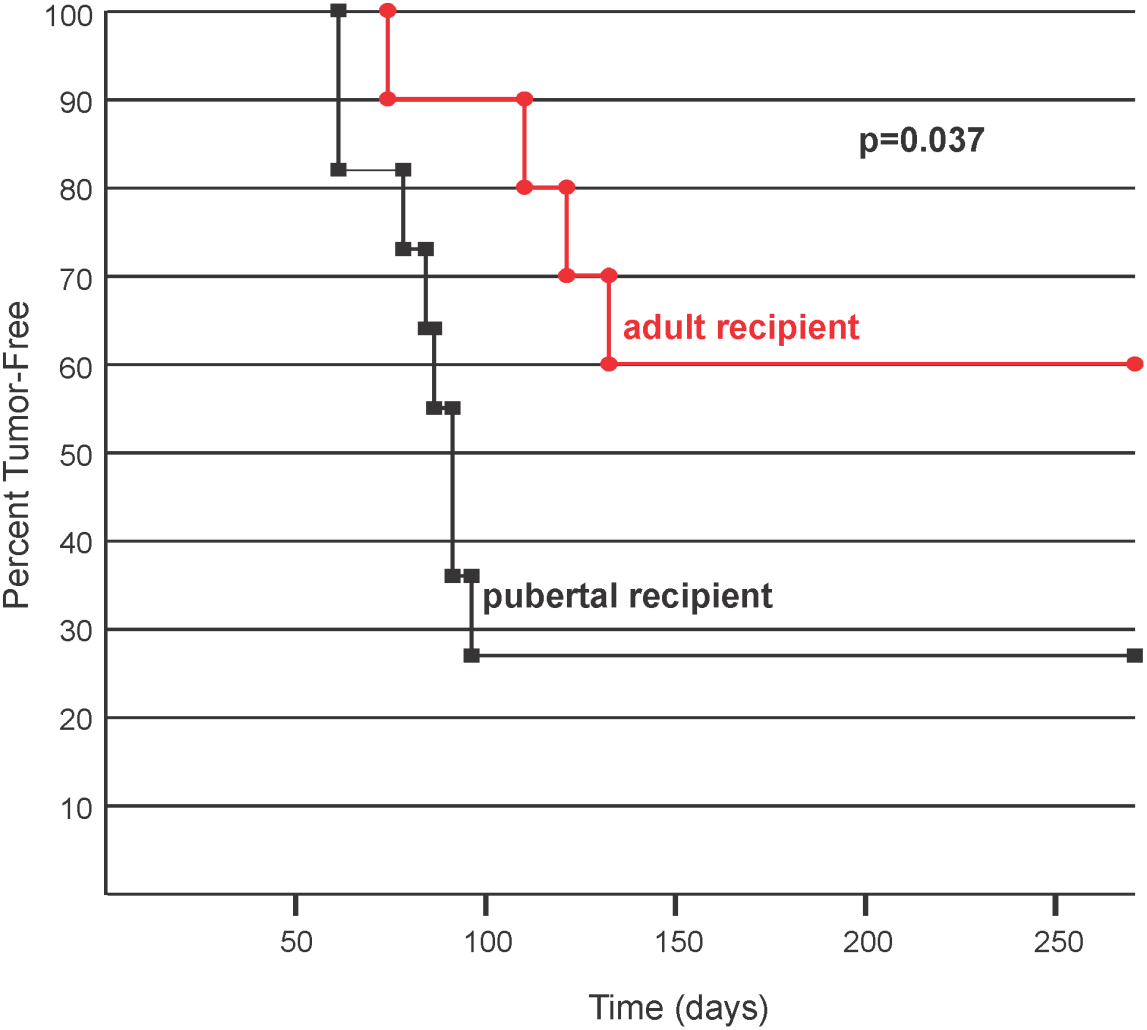
Kaplan-Meier plot showing the percentage of tumor-free mice in which mammary epithelial cells from 55 day old MTB-IGFIR mice were transplanted into pubertal (black line) or adult (red line) recipient mice. Tumor onset was significantly faster in pubertal recipients as determined by Breslow (Generalized Wilcoxon) analysis.

**Figure 6:**
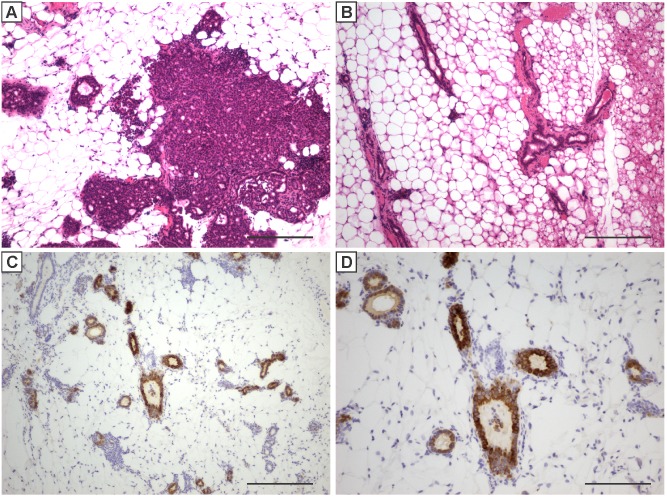
Representative H&E stained sections of hyperplasia (A) and normal mammary ducts (B) that developed following transplantation of pubertal MTB-IGFIR mammary epithelial cells that did not progress to palpable tumors. Immunostaining for IGF-IR (C, D) revealed high levels of expression with normal mammary ducts indicating successful engraftment of transgenic tissue. Scale bars, 200 µM (A–C) and 100 µM (D).

The decreased tumor latency associated with IGF-IR transgene expression during the pubertal phase could be due to the presence of an increased progenitor cell population and/or the influence of the hormonal and stromal environment. To address this, mammary tissue containing epithelial cells from doxycycline naïve, pubertal MTB-IGFIR mice were transplanted into cleared mammary fat pads of 40 day old (pubertal) or 124 day old (adult) recipient mice. Engrafted mice were immediately placed on doxycycline supplemented food (to induce the IGF-IR transgene) and mice were monitored for tumor development. As shown in Figure 5 and Table 2, mammary tumor onset was significantly shorter following transplantation into pubertal cleared mammary fat pads compared to the adult cleared fat pads. Tumor incidence was also higher in pubertal recipient mice compared to adult recipient mice (Table 2). Two of the pubertal recipient mice and a single adult recipient mouse that did not develop palpable mammary tumors contained regions of hyperplasia that were visible upon histologic examination (Fig. 6A). Therefore, 91% of the pubertal recipient mice and only 50% of the adult recipient mice developed mammary tumors or hyperplasia (Table 2). Cleared mammary fat pads that did not develop tumors contained epithelial ducts with normal architecture (Fig. 6B) comprised of mammary epithelial cells expressing high levels of IGF-IR (Fig. 6 C, D). The presence of IGF-IR positive epithelial cells in recipient mice indicates that engraftment with MTB-IGFIR epithelium was successful in all mice. There were no obvious histological differences between tumors that developed in the 40 day old or 124 day old recipient mice and none of the transplant mice developed lung metastases.

**Table 2:** Tumor Characteristics of 55 Day-Old Epithelium Transplanted at Day 40 or Day 124.

	Transplant at Day 40	Transplant at Day 124
Number of Mice with Tumor	8/11 (73%)	4/10 (40%)
Tumor Onset	81.0±4.8	109.2±12.6
Tumor Specific Growth Rate	3.69±0.11	3.45±0.16
Number of Mice with Tumor or Hyperplasia	10/11 (91%)	5/10 (50%)

## Discussion

One of the greatest risk factors for most cancers is age. As individuals age, their chances of developing many common types of cancer such as lung, breast, colon, and prostate, increase dramatically as the individual increases with age [36]. There are exceptions to these generalizations as hematopoietic and CNS malignancies can occur in children [37]. One explanation for the association between age and cancer incidence is that cancers result from an accumulation of mutations that occur over the course of years or decades. One clear example of this lag between tumor initiation and cancer development is lung cancer resulting from cigarette smoking. The popularity of cigarette smoking increased in the 1940s however, lung cancer rates did not begin to steeply rise until the 1970s [38]. Cigarette smoking peaked in the US in the mid-1960s and began to decline thereafter, however, lung cancer rates did not begin to decline until the 1990s [38]. This time lag between tumor initiation and cancer development makes it difficult to define initiating events and hinders research into cancer prevention. Research on breast cancer prevention is further complicated by the fact that unlike most organs, developmental changes in the mammary gland occur primarily during postnatal life [2], [6], [39], [40].

A number of human and rodent studies have shown that exposure of the mammary gland to chemical carcinogens, radiation or endocrine disrupting compounds during embryonic or pubertal development can enhance the susceptibility of the mammary gland to tumor development while completion of a full term pregnancy protects against tumor development [8], [19], [41], [42]. To determine whether oncogenic transformation of mammary epithelial cells displayed similar differential susceptibility based on developmental stage, the expression of the IGF-IR transgene was induced during embryonic development, prior to pubertal onset, in young adult, virgin mice, and after a full term pregnancy. Using this strategy, it was shown that mammary epithelial cells were most susceptible to oncogenic transformation when the IGF-IR transgene was expressed during pubertal development. Tumor latency was significantly shorter and tumor multiplicity was significantly greater following pubertal IGF-IR transgene expression compared to adult or primiparous IGF-IR expression. Thus, the mammary gland is particularly susceptible to oncogene-induced transformation of mammary epithelial cells during pubertal development and pregnancy provides some protection against tumor development.

One main difference in the protective effect imparted by pregnancy in our study was that mammary tumor development was delayed but still occurred in 100% of the mice. In contrast, studies administering carcinogens to rats following pregnancy typically observed a decrease in mammary tumor incidence [43]–[45]. One possible explanation is that IGF-IR is a more potent transforming agent than the carcinogens. This is likely considering carcinogen administration did not always produce tumors in 100% of the pubertal mice [43]–[45]. It is also possible that the timing of the oncogenic stimuli altered tumor incidence since the IGF-IR was continually expressed after the full term pregnancy while carcinogens were typically administered as a single dose or multiple doses over 4–6 weeks.

In this study we showed that the mammary gland is particularly susceptible to transformation during pubertal development. Two human studies support this finding. The first study showed that Japanese women who were under 20 at the time of the atomic bombing of Hiroshima and Nagasaki have a 2-fold increased lifetime risk of developing breast cancer compared to women who were 35 years of age or older at the time of radiation exposure [12]. The second study evaluated standardized morbidity ratios (SMR: ratio of the number of women in the study that died of breast cancer compared to the expected number of deaths in the population) for women treated with supradiaphragmatic irradiation for Hodgkin’s lymphoma at different ages. Women who were 14 years old or younger at the time of radiotherapy had a SMR of 279 while those who were 15–19 years old or those who were over the age of 30 had SMRs of 74.5 and 4.6 respectively. These breast cancers took at least 15 years to develop after treatment [16].

Interestingly, induction of the IGF-IR transgene during embryonic development did not reduce tumor latency compared to induction of the IGF-IR transgene during pubertal development. If mammary tumor onset was presented as postnatal age rather than days of doxycycline exposure, tumor onset for the embryonic mice was 74±2.1 days of age while tumor onset for the pubertal mice was 74±2.5 days of age. Tumor multiplicity was also similar in the two groups. The only apparent difference between embryonic and pubertal IGF-IR overexpression was a non-significant increase in the number of metastatic lung lesions in the mice with embryonic IGF-IR overexpression. Thus, our data suggests that oncogene exposure in mammary epithelial cells during embryonic development does not enhance mammary tumor development more than oncogene exposure during pubertal development. This finding is somewhat surprising considering studies exposing rodent embryos to endocrine disruptors found that these mice were more susceptible to mammary tumor development as adults [46], [47]. In addition, humans exposed to the synthetic estrogen diethylstibestrol (DES) during fetal development had increased breast cancer rates [48]–[52]. It is possible that systemic administration of endocrine disruptors modulate the hormonal or stromal environment of the mammary gland rendering it more susceptible to transformation. In our study, IGF-IR transgene expression was restricted to the mammary epithelial cells and thus should not significantly impact the stromal or hormonal environment of the mammary gland.

Our mammary transplant studies highlight the importance of the microenvironment in tumorigenesis. By engrafting the same tissue into mammary glands at different developmental stages, we observed that overexpression of the IGF-IR transgene in mammary epithelial cells produced mammary tumors more frequently and faster when engrafted into a pubertal mammary microenvironment compared to an adult mammary microenvironment. Thus, circulating hormones and/or the production of specific factors by mammary stromal cells during puberty create conditions conducive for epithelial transformation.

In conclusion, our data shows that the mammary gland is particularly susceptible to transformation during pubertal development and the stromal/hormonal environment associated with this developmental stage dramatically influences the susceptibility of the mammary gland to transformation. Defining the stromal and hormonal differences between the adult and pubertal mammary gland may produce strategies to reduce transformation during this particularly sensitive developmental window.
